# Social Feedback and the Emergence of Rank in Animal Society

**DOI:** 10.1371/journal.pcbi.1004411

**Published:** 2015-09-10

**Authors:** Elizabeth A. Hobson, Simon DeDeo

**Affiliations:** Pennsylvania State University, UNITED STATES; 1 National Institute for Mathematical and Biological Synthesis, University of Tennessee, Knoxville, Tennessee, United States of America; 2 Department of Biology, New Mexico State University, Las Cruces, New Mexico, United States of America; 3 Department of Informatics, School of Informatics and Computing, Indiana University, Bloomington, Indiana, United States of America; 4 Santa Fe Institute, Santa Fe, New Mexico, United States of America

## Abstract

Dominance hierarchies are group-level properties that emerge from the aggression of individuals. Although individuals can gain critical benefits from their position in a hierarchy, we do not understand how real-world hierarchies form. Nor do we understand what signals and decision-rules individuals use to construct and maintain hierarchies in the absence of simple cues such as size or spatial location. A study of conflict in two groups of captive monk parakeets (*Myiopsitta monachus*) found that a transition to large-scale order in aggression occurred in newly-formed groups after one week, with individuals thereafter preferring to direct aggression more frequently against those nearby in rank. We consider two cognitive mechanisms underlying the emergence of this order: inference based on overall levels of aggression, or on subsets of the aggression network. Both mechanisms were predictive of individual decisions to aggress, but observed patterns were better explained by rank inference through subsets of the aggression network. Based on these results, we present a new theory, of a feedback loop between knowledge of rank and consequent behavior. This loop explains the transition to strategic aggression and the formation and persistence of dominance hierarchies in groups capable of both social memory and inference.

## Author Summary

An individual’s success depends critically on socially-constructed properties such as rank. A detailed study of two independent captive parakeet groups reveals how these properties come into being. We show that individuals can use localized patterns in the aggression network to learn the relative ranks of individuals, and that these signals of rank strongly correlate with individual decisions to aggress. Over time, feedback between knowledge and behavior leads to the emergence of strategic aggression: individuals focus their aggression on those nearby in rank.

## Introduction

Individuals from social species must interact with each other to reproduce, find food, and survive. Higher-level social structures such as hierarchies emerge when interacting individuals need to manage trade-offs in the costs and benefits of social associations [1, 2]. One of the most important is the dominance hierarchy, where group-wide “global” rankings are derived from local aggressive interactions, and form emergent social properties [3–5].

Aggression has obvious immediate costs, including energy expended and the possibility of injury. Benefits, conversely, can be both immediate and delayed. Individuals may fight to gain immediate access to contested resources, or they may aggress in order to gain rank, which then provides these individuals with delayed rank-dependent benefits. Aggression that results in higher dominance rank often increases an individual’s access to foraging resources and reproductive opportunities (*e.g.*, Ref. [6–8]).

Groups across a broad range of taxa are structured by dominance rank [9] despite large variation in cognitive skills. Dominance hierarchies are found in primates [10, 11], social carnivores [12, 13], ungulates [14, 15], birds [16–19], fish [20], and even crustaceans [21, 22] and insects [23]. These group-level social structures form and stabilize on the basis of perceptions and actions necessarily made at the individual level [24]. Dominance rank is generally achieved through a series of aggressive events, and hierarchy formation takes place without top-down control in a manner that is largely independent of the intrinsic properties of the individuals involved [25–27].

Previous experimental and theoretical work on dominance hierarchies shows that interaction outcomes can shape how individuals behave in subsequent interactions [28], that individuals may use a jigsaw approach to determine how to interact with a few individuals based on observations of event outcomes [25, 29], and that observed win/loss outcomes can be strong determinants of an individual’s choice of targets of future aggression [30–32]. In some systems, “badges of status”, or conventional signals, can enable individuals to estimate the rank of others [33, 34]. However, these visual signals of rank can differ in their prevalence within species under different conditions (*e.g.* [35] and [36]), and may only explain part of the dynamics of dominance hierarchy formation (*e.g.* [37]). Aggression preferences of individuals in some groups can also be driven by spatial patterns, especially in cases where closely-ranked individuals are more likely to be in spatial proximity [38–41]. However, little is known about rank formation in groups where these simpler behavioral rules or cues to rank are absent.

Recent evidence suggests that individuals in many species have the cognitive ability to use their observations of social interactions of others to inform their own behaviors, including several primate species [10, 11, 42–44], ravens [45], hyenas [46], and fish [47–50]. However, while this evidence shows that individuals observe each other and react to these observations, we do not currently understand how individuals integrate information on the outcomes of their own interactions, with observations of others’ interactions, in order to determine rank.

We are thus faced with two distinct questions. How does rank become relevant to individual decisions to aggress? And, what information or mechanism might individuals use in order to learn emergent social properties like rank? This paper presents three findings, based on detailed, highly-resolved observations of aggression in a social avian species, that answer these questions by reference to the interaction between local decision-making and global system properties.

Our first finding is a strong signal of the influence of rank on decision-making. This is seen in how aggression is allocated: target choice became structured over and above what is necessary to reproduce the rank order alone. This structuring happened in a manner that could not be accounted for by individual characteristics, or by the spatial position of individuals. In both groups, it occurred around a week after initial group formation. After this transition, individuals preferentially directed aggression more frequently towards those nearby in rank and avoided interactions with those far below them in rank.

Our second finding is that in this structured society, both levels of aggression and subsets of the full network (network motifs in the form of aggression chains) provided cognitively-accessible signals of rank. These pathways are the likely mechanisms through which rank is inferred. Rank is a global property, but in these structured systems can be learned by judicious observation of local interactions.

Our third finding is that the motif pathway not only provided a signal of relative rank, but was strongly predictive of actual behavior. Individuals were far less likely to direct aggression against the terminal individual in an observable aggression chain.

Taken together, these results help explain the emergence of rank as an interaction between two processes: inference of rank from cognitively-accessible social signals, and decision-making that correlates with these signals. They indicate a critical role played by a knowledge-behavior feedback loop—between inference of group-level properties, and consequent decision-making. Such feedbacks may be a critical pathway for how evolved systems reduce uncertainty by tying together multiple timescales [51]; our findings here have parallels in discoveries of how signal use in primates tracks coarse-grained features of a social network [24, 52].

These results provide new insight into the problem of choosing targets and establishing a dominance hierarchy in species that lack simple perceptual cues, such as size or spatial location, for an individual’s rank. In these more complex societies, rank order is necessarily a cognitive construct that summarizes the many dyadic-level interactions into an emergent group-level property.

Our results derive from studies of two independent groups of monk parakeets (*Myiopsitta monachus*), a small neotropical parrot native to temperate South America and notable for its highly social colonial and communal nesting behavior [53] as well as its widespread success as an invasive species [54–57]. Monk parakeets exhibit several characteristics of complex sociality [58–60], and to our knowledge, is the first parrot species in which detailed and quantitative dominance hierarchy analysis has been conducted [58].

Studies of dominance hierarchies in parrot species present an intriguing comparison to those conducted on primates and humans. Parrots share many characteristics with primates, such as large relative brain size and advanced cognition [61, 62], extended developmental period [61, 63], long lifespans [64–66], and individualized relationships within complex social groups [58, 67]. Additional characteristics, such as vocal learning and high fission-fusion dynamics, are uncommon in most primates [68], but are shared by parrots and humans [69]. Understanding how parrots form and maintain dominance relationships in complex social groups thus has the potential to further our more general understanding of rank in socially and cognitively complex species.

## Methods

### Ethics statement

All animal activities conducted during this study were approved by the New Mexico State University Institutional Animal Care and Use Committee (protocol number 2006-027).

### Data collection protocols

Our study is based on observation of directed aggression in groups of monk parakeets housed in captivity at the U. S. Department of Agriculture National Wildlife Research Center in Gainesville, Florida. We formed two independent groups (*N* = 21 and 19) and observed aggressive events during novel group formation (additional details in Refs. [58–60]). Prior to our study, parakeets were housed in smaller cages (median group size = 2); while some were in visual contact, direct physical contact between individuals in different cages was not possible. Random group assignment resulted in the formation of social groups largely comprised of novel dyadic associations: only 3% (Group One) and 6% (Group Two) of dyads were composed of birds housed together during the 8 months preceding the study [59]. To facilitate individual identification, we marked each bird with a unique facial pattern using colored nontoxic permanent markers (Sharpie, Inc.).

Each captive group was released sequentially into a 2025 m^2^ semi-natural outdoor flight pen and observed over the course of 24 days by 1–4 observers. We used all occurrence sampling [70] to record data on directed agonistic behaviors. As in Ref. [58, 60], we restricted our analysis to dyadic aggression where events had clear outcomes. We focused on intentional aggressive behavior, which we define here as events where one individual (the actor) approached another (the target) and the overt aggressive actions of the actor caused the target to be physically displaced and supplanted from its perch by the actor. This resulted in a win for the actor and a loss for the target of the aggression. Because some actors aggressed against targets in a string of frequent sub-attacks (*e.g.* 6 attacks in 10 sec), we required a cessation of aggression from the actor toward the target of at least 60 sec in order to define the aggression as a clear win; thus an actor could only achieve a full win once against a target per minute. We do not include more subtle forms of aggressive signaling, nor aggression that occurred during scramble competition, because specificity of the direction of aggression was less clear and overt aggression was often reactive rather than intentional in these cases. We hereafter refer to these intentional, dyadic, aggressive wins as ‘aggressive wins’ or ‘aggression’.

We divided the 24-day study period into four 6-day study quarters to facilitate comparisons across the two replicate social groups.

### Estimation of dominance hierarchies

Keeping track of the total number of aggressive events between any two individuals allows us to define the directed aggression network; *d*_*ij*_, the number of observed aggressive events directed by *i* against *j*. We then use Eigenvector Centrality (EC, Ref. [71]) on the directed aggression network as our primary means of determining rank. EC assigns a centrality score, *v*_*i*_, to each individual *i*, using both the direct and indirect links in aggression networks in a recursive fashion. High centrality then equates to low power. Intuitively, an individual has low power if it is the recipient of many aggressive events from those who themselves have low power. Once we have observed *d*_*ij*_, we can then define the normalized aggression, *t*_*ij*_, as

$$\begin{array}{c} t_{ij}=\frac{d_{ij}+\epsilon }{\sum_{k=1}^{N}\left(d_{ik}+\epsilon \right)}, \end{array}$$ (1)

where *ϵ* here is a small regularizing term. Then, the centrality scores for the individuals in the system, *v*_*i*_, are those that satisfy

$$\begin{array}{c} v_{i}=\sum_{j=1}^{N}t_{ij}v_{j} \end{array}$$ (2)

EC is one of the main algorithms for determining consensus beliefs within a network [72], and dominance ranks based on EC power scores are strongly correlated with the main alternative ranking methods such as I&SI [73]. A benefit of EC over pure ranking methods is that EC allows for the direct quantification of power, rather than just a linear order; this means it can distinguish cases where two individuals are “nearly equivalent” in rank from those where differences in dominance are reliable and large. Both the bootstrap error estimates we report, and the null models we use, preserve these distinctions: they reproduce the underlying power scores, rather than just the rank difference.

EC is closely related to David’s Score (DS; Ref. [74]). In contrast to EC, which uses all the interactions in the aggression network to define the status of a particular individual, DS only includes interactions up to two steps away (my aggressors’ aggressors; my targets’ targets). Both DS and EC have found widespread use in the characterization of rank in animal groups [24, 52, 60, 73, 75–78].

EC and DS are both “depth” methods [75]. They quantify rank by reference to network properties, and weight the interactions between two individuals *i* and *j* in ways that depend on interactions each individual has had with others.

Conversely, measures like Weighted Simple Consensus (WSC) are “breadth” methods [75], in which rank is estimated based only on the interactions directed towards individual *i*. For example, WSC estimates rank as the product of the total number of an individual’s aggressors and the total amount of incoming aggression [75]. Ref. [75] found EC comparable to WSC, both of which performed well. In our data, EC correlates strongly with all three measures (I&SI, DS, WSC); see S1 Table.

### Average rank aggression

We are most interested in how relative rank influences how and where individuals direct their aggression. To measure this, we use average rank aggression, *R*(Δ). *R*(Δ) is the amount of individual-level aggression, per unit time, directed at those Δ ranks away. It is defined as:

$$\begin{array}{c} R\left(\Delta \right)=\frac{1}{N_{\Delta }T_{\text{obs}}}\sum_{i=1}^{N}d_{i\Delta } \end{array}$$ (3)

where *d*_*i*Δ_ is the amount of aggression directed by *i* at the individual whose rank is Δ steps away; *N*_Δ_, the total number of individuals who have a potential target Δ ranks away; and *T*_obs_ the total observation time. A rank lower than *i*, *i.e.*, “down” the hierarchy, is indicated by Δ greater than zero; a rank higher than *i* is indicated by Δ less than zero.

Average rank aggression is our primary signal of individual decision-making. We are interested in determining whether the observed aggression indicates the influence of rank on decision-making. In order to do this, we construct null models for the range of behaviors we expect to see if individuals interact in a unstructured fashion. We explore a related measure, average preferential rank aggression, in S1 Text.

### Null models for aggression

A critical step in our analyses is determining when and how patterns of aggression differ from what might be expected from random noise in otherwise unstructured behavior. To do this, we construct a hierarchy-constrained null model. This null model produces aggression networks that reproduce the observed power scores, without imposing particular rules about who directs aggression against whom. Such a model is possible because in a group of *n* individuals there are *n*(*n*−1) free parameters in an aggression network, but only *n*−1 numbers are required to specify that network’s EC power scores.

EC (indeed, any ranking or scoring system) thus amounts to a lossy compression of the original data [79], summarizing the behavioral patterns relevant to the establishment of a dominance hierarchy. Conversely, for any given dominance hierarchy there are many possible behavioral patterns. In particular, because there are many possible **d** and **t** matrices compatible with a particular power distribution *v*, we can define a null model as random draws from the set of matrices that have, on average, the same *v*. Our null model for aggression is defined as a random sample from this larger set; we also preserve the total aggression of each individual (additional details in S2 Text).

Any particular sample from this null will preserve (on average) the EC power scores, but will be otherwise unstructured and contain no correlations that are unnecessary to preserve those scores. We can measure relevant properties, such as *R*(Δ), on these null networks. Deviations in the real data from these nulls indicate individuals are systematically directing aggression in ways that differ from what is otherwise expected for an aggression network with that dominance structure.

### Simpler signals of rank

We investigated whether parakeets could use size characteristics or spatial positioning, rather than social observations, to infer rank. We measured several morphometric characteristics which reflect body size: wing chord, culmen depth, culmen width, and mass. We used these data to determine whether rank could be predicted by an individual’s size. We also collected data on spatial patterns. As in [58, 60], we determined the identity of each individual’s nearest neighbor (within 10m^2^ quadrats) during scan samples to determine whether rank affected spatial association patterns. If birds nearby in rank tend to spend more time physically near each other, spatial proximity could serve as a signal of rank. In particular, we would expect a negative correlation between (absolute) relative rank difference between *i* and *j*, and the number of times *i* was observed to be *j*’s nearest neighbor in space.

### Social signals of rank

Estimating rank via social observations and signals can be computationally challenging. As noted by Ref. [75], breadth measures such as WSC are of particular interest because they correlate with the more sophisticated depth measures, but are more likely to be cognitively-accessible to individuals within the system. For this reason, we also measure WSC on the directed aggression network. The WSC score of an individual is the product of the total amount of aggression (number of events) received, multiplied by the number of distinct individuals who directed that aggression (additional details in S4 Text). We order individuals by these dominance scores to determine individual rank. As in the case of EC, a higher WSC equates to lower rank because these individuals receive more aggression from more individuals.

Individuals may use breadth-based signals such as WSC, but they may also use measures sensitive to other features of the network. In particular, even though EC rank is a property of the aggression network as a whole, small portions of that network may contain signals of relative rank, and these smaller network subsets may be more easily perceived.

To study information of this second kind, we focus on a particular kind of motif—the aggression chain—where we can trace a line of aggression from individual *i*, to individual *j*, to individual *k*, and so forth. We measure the signals contained in such chains using average weighted rank difference, *W*(*n*). *W*(*n*) quantifies the extent to which an aggression chain provides information about relative rank difference for chain length *n*. *W*(2) is defined as

$$\begin{array}{c} W\left(2\right)=\frac{\sum_{i,j,k;\emptyset }d_{ij}d_{jk}\Delta \left(i,k\right)}{\sum_{i,j,k;\emptyset }d_{ij}d_{jk}}, \end{array}$$ (4)

where Δ(*i*,*k*) is the rank difference between *i* and *k*, and ∅ indicates that in any instance, the identities of individuals *i*, *j*, and *k* do not overlap. *W*(2) is then the average rank difference between any two individuals, weighted by the product of aggression seen along all chains connecting them.

*W*(2) takes into account not only the existence of the chain, but also its strength. In general, weighted network ties are generally more informative about the social relationships among individuals and are more robust to sampling differences [59, 80–82] than relying solely on presence-absence information. *W*(*n*) for *n* larger than two is defined similarly (see S3 Text); the number of possible motifs we need to examine to compute *W*(*n*) grows exponentially with depth, and we stop our analysis at *n* equal to six. (Note that while the total number of motifs grows exponentially with *n*, *W*(*n*) quantifies the average amount of information in any particular aggression chain, not the total amount of information in all chains.)

When *W*(*n*) is significantly different from the null, this indicates the *presence* of information in aggression chains over and above what is expected from systems with the same power scores, but otherwise unstructured aggression.

### Use of motif-based signals of rank

To determine if individuals actually perceived and used these motifs as a signal of rank, we measure the behavioral signatures of transitive inference. Transitive inference occurs when one individual uses knowledge about its own interactions with a target (*j*) and third-party observations of how *j* interacts with additional targets (*k*, *l*, *m*, …) to infer its own likelihood of winning over one of *j*’s targets, such as *k*. For the case of three individuals, *i* and *k* would have a transitive relationship with individual *j* if the amount of aggression directed from *i* to *j* (*d*_*ij*_) and the aggression from *j* to *k* (*d*_*jk*_) was related to the amount of aggression *i* directed to *k* (*d*_*ik*_).

**Fig 1:**
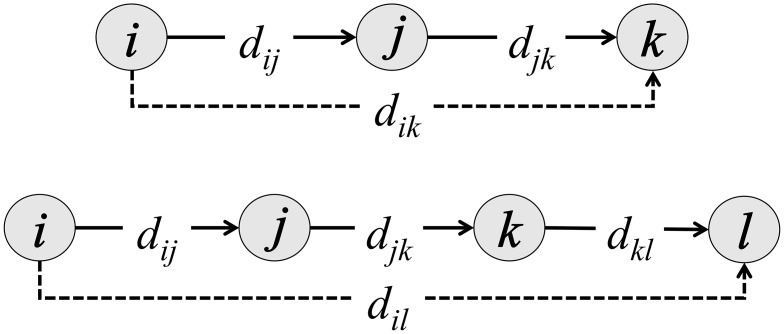
Illustration of aggression chains of length two and three. The *d* terms indicate the number of aggressive events between individuals *i*-*l*. The originator of the chain (*i*) is then linked through these indirect interactions to the terminal individual (*k* or *l*), and the dashed line indicates the association between the originator and the terminal individuals in the chain. For a chain length of two, *W*(2) quantifies the extent to which aggression along the chain (*d*_*ij*_ and *d*_*jk*_) is a signal of rank difference between the originator and the terminal individual (dashed line); *T*(2) quantifies the extent to which aggression directed from the originator *i* to the terminal individual (dashed line) is higher, or lower, than average aggression by *i*, given aggression levels within the chain *d*_*ij*_ and *d*_*jk*_.

We tested for transitive relationships between individuals in the first and last positions anchoring each aggression chain (Fig 1), up to a chain length of 6. We quantified fractional transitivity, *T*(*n*), for a chain length of two as:

$$\begin{array}{c} T\left(2\right)=\frac{\sum_{i,j,k;\emptyset }d_{ij}d_{jk}\left(d_{ik}-{\bar{d}}_{i;j}\right)/{\bar{d}}_{i;j}}{\sum_{i,j,k;\emptyset }d_{ij}d_{jk}} \end{array}$$ (5)

where ${\bar{d}}_{i;j}$ is the average aggression *i* directed towards all individuals other than *j*, and ∅ indicates that our sums exclude cases where the identities of individuals *i*, *j*, and *k* overlap. The natural extension to *n*-step chains, *T*(*n*), is defined in S3 Text. *T*(*n*) is positive or negative when an individual *i* increases or decreases, respectively, its aggression against *k* given that *k* is at the end of an *n*-step chain.

Very positive *T*(*n*) means that individuals prefer to aggress against those at the end of chains while very negative *T*(*n*) means that individuals preferentially avoid such aggression. When *T*(*n*) is significantly different from null, this indicates the *use* of information in aggression chains over and above what is expected from systems with the same power scores, but otherwise unstructured aggression.

### Data exclusions

Our initial analysis indicated that differences in aggression patterns in Group One and Group Two were strongly driven by a single individual, NBB, in Group Two. This individual was persistent in her attempts to affiliate with others in Group Two, but was not able to form a strong affiliative relationship within the group [58]. Aggression directed at NBB appeared to be mostly reactive aggression in response to NBB’s persistent and apparently unwanted attempts to affiliate rather than intentional target selection choices by actors choosing to attack NBB.

Because our focus with this work was on intentional target selection and strategic aggression, and because the actions of NBB were anomalous compared to all the other individuals in the group, we excluded NBB from the main analyses. However, we present the results for Group Two including NBB in the Supplementary Information (S1 Fig and S2 Fig) to show how unusual decision-making by this individual affects the overall patterns created by the other 18 individuals.

## Results

We analyzed a total of 1013 aggressive wins in Group One and 1360 wins in Group Two over the 24-day study periods. Despite the captive conditions, some individuals avoided interacting which each other, resulting in incompletely connected aggression networks in both groups (density of 0.89 in Group One; 0.92 in Group Two).

### Simpler signals of rank

We find no evidence that rank could be reliably determined based on simple underlying cues such as size or spatial proximity. None of the morphometric body size measures provided any rank signal in either of the two study groups. Rank was not significantly associated with the physical size of individuals, including weight, wing length, and beak properties (∣*r* ^2^∣ < 0.18, *p* > 0.05, S3 Fig).

Nor did we find that spatial proximity provided a signal of rank (S4 Fig). Neighbor identity is not a strong signal of rank in either Group One (*r* ^2^ = −0.12) or Group Two (*r* ^2^ = −0.02). Even in the final three-quarters, where behavior is most regular, in Group One, proximity provides only a weakly anticorrelated signal (*r* ^2^ = −0.14); in Group Two, it provides no signal at all (*r* ^2^ = 0.04; consistent with null).

### Emergence of social structure

**Fig 2:**
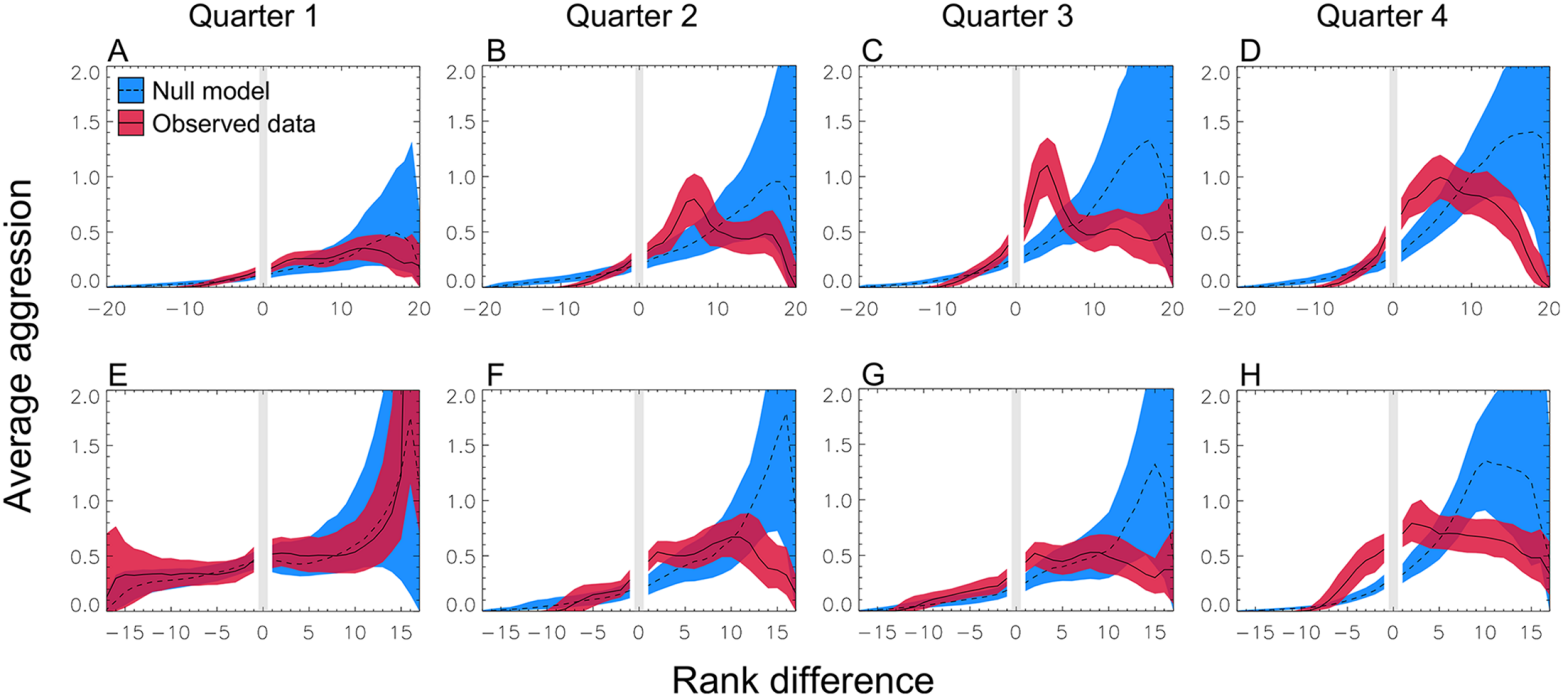
The emergence of structured aggression. Average rank aggression (*R*(Δ), Eq 3) for Group One (a-d) and Group Two (e-h) across study quarters; each quarter is six days long. Units of *y*-axis are aggressive acts per individual per 1000 minutes (100 scan periods). Patterns of aggression (red band/solid line) in both groups are consistent with the null (dashed line/blue band) in the first Quarter, but diverge in Quarters two, three and four as individuals focus their aggression on those nearby in rank. Points are averaged ±1 rank; bands are 1*σ* bootstrap-estimated error ranges. Maximum rank difference in Group Two (*N* = 19) is 17 because one individual was dropped.

Average rank aggression in both Groups One and Two was consistent with the null model during the first quarter of the study period (Fig 2a and 2e). However, behaviors quickly diverged from null expectations as individuals began to structure their behavior in ways strongly correlated with rank. Aggression patterns in the final three-quarters of the study period all diverged strongly from null expectations (Fig 2b–2d and 2f–2h), with aggression strongest towards individuals nearby in rank.

**Fig 3:**
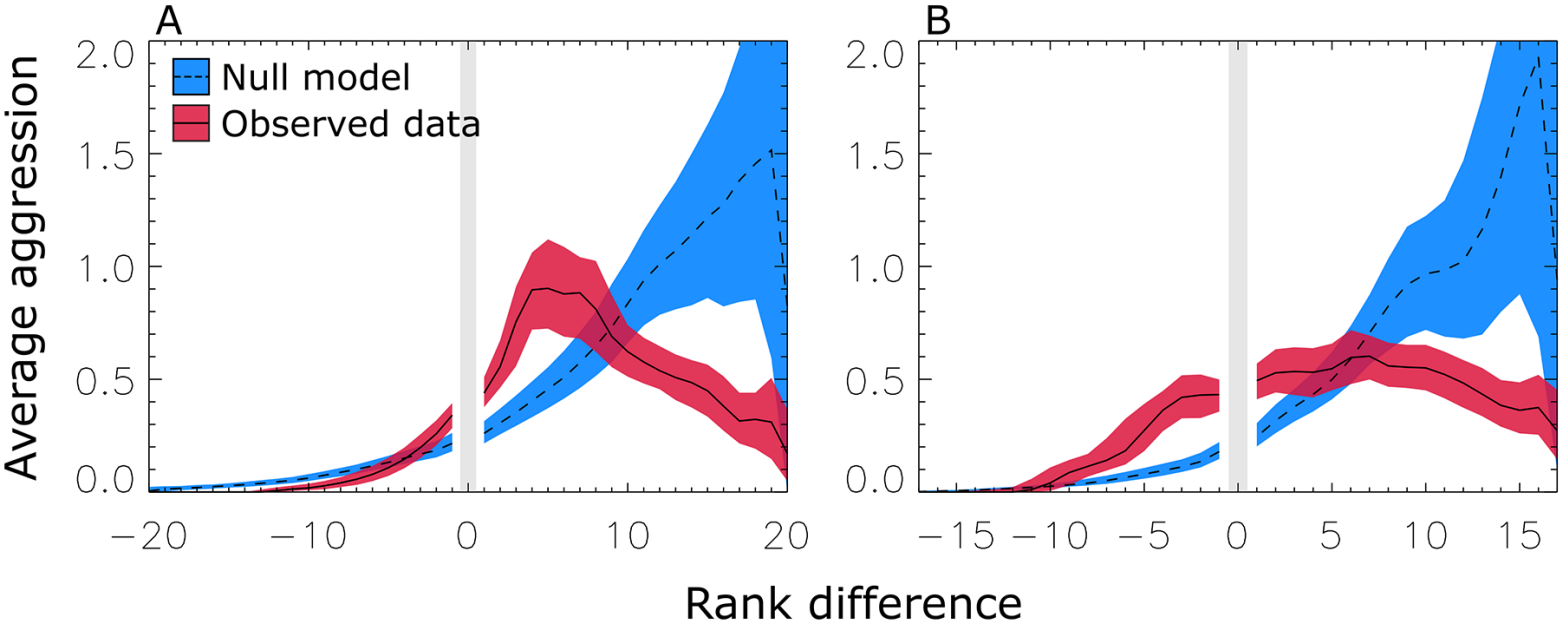
Structured aggression. Preferential rank aggression (*R*(Δ), Eq 3) pooled over the final 3 quarters of the study period for Group One (a) and Group Two (b). Pooling data provides a gain of signal-to-noise that further refines our understanding of the strategies at work once the behavior-knowledge feedback is in place. Both groups show a focusing of aggression towards those nearby in rank, over and above null expectation. Higher levels of rank entrepreneurship—aggression directed, contrary to expectation, upwards in the hierarchy—are visible in Group Two. Points are averaged ±1 rank; bands are 1*σ* bootstrap-estimated error ranges.

We refer to this as rank-focused aggression. Combining the final three quarters of the data increases this signal (Fig 3a and 3b). We can quantify the emergence of this phenomenon by reference to the ratio of null to observed aggression for nearby ranks (one to five steps below). While neither group showed above-null aggression to nearby ranks in quarter one (*p* > 0.05), both showed significant deviations and large effect sizes for later quarters. Group One had aggression at 49% above-null (*p* < 0.05) in the second quarter; and a factor of 2.7 and 2.2 times higher in quarters three and four (*p* < 0.001). Group Two had aggression at 60% above null (*p* < 0.05) in the second quarter, and 64% and 56% in quarters three and four (*p* < 0.05). Aggregating over the final three quarters gives an overall signal of rank-focused aggression of a factor of 2.1 times above null (Group One, *p* < 0.001) and 51% above null (Group Two; *p* < 0.01). We find equivalent results for a related measure, average preferential rank aggression; see S5 Fig.

This is our first main result. The onset of strong deviations from the nulls points to the emergence of structured behavior at the individual level. The structure of this behavior is dictated, at least in part, by relative rank.

### Inferring rank: The magnitude pathway

Determination of rank via depth-based measures is computationally intensive. In our data, depth-based EC correlated strongly with breadth-based WSC (*r* ^2^ ≈ 0.73 in both groups) and thus knowledge of WSC can provide at least partial knowledge of EC. Because of WSC’s reliance on levels of aggression alone, rather than network structure, we refer to this rank-inference mechanism as the “magnitude pathway”.

We look for signals of the use of this pathway by considering evidence that aggression is structured as a function of relative WSC rank. We find evidence for reduced aggression at individuals widely separated in WSC rank (large positive Δ) compared to the null. This indicates that, in addition to providing knowledge of rank, WSC-derived rank is also predictive of some features of individual aggression. However, we do not see strong evidence for increased aggression to those nearby in WSC rank—none in Group One, and only weak evidence in Group Two (S6 Fig).

The fact that WSC was predictive of aggression indicates that the magnitude pathway may play an important a role in structuring aggression. However, the absence of a signal of rank-focused aggression implies that there were patterns of aggression *invisible* to the breadth-based WSC measure. If individuals used WSC signals to help direct their aggression to those nearby in rank, they must have been supplementing them with other sources of information. The absence of rank-focused aggression in the WSC case is an example of how rank defined by reference to WSC does not capture all of the structure relevant to individual aggression. This provides an implicit justification for the decision, made earlier, to use EC as the primary measure of rank in the system.

### Inferring rank: The motif pathway

**Fig 4:**
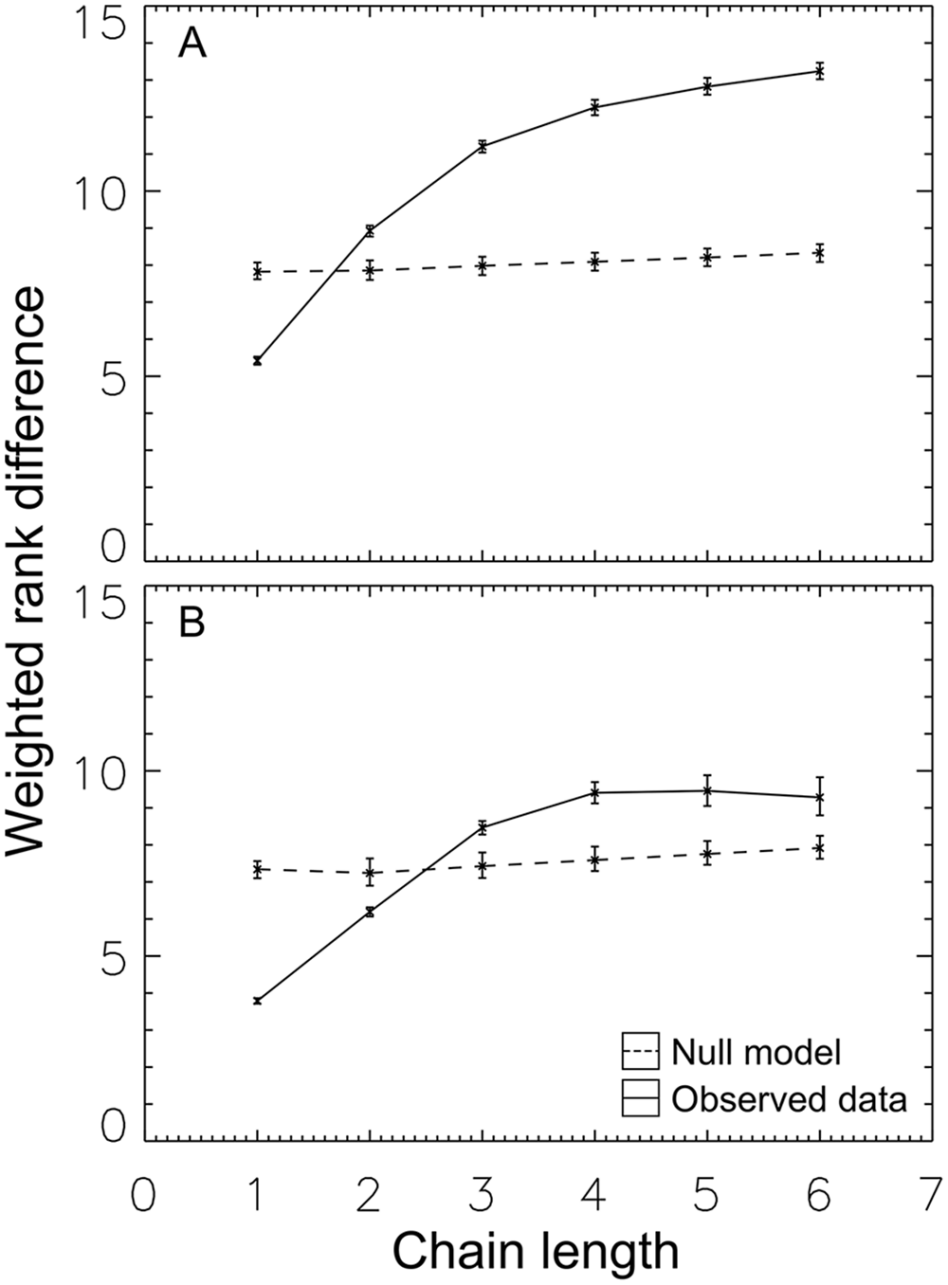
Cognitively-accessible social properties. Shown is the weighted average rank difference for different aggression chain lengths (*W*(*n*), Eq 4) for Group One (a) and Group Two (b). The solid line shows the observed signal, while the dashed line shows that found in the null, with 1*σ* error ranges. Significant evidence exists for a strong signal of relative rank contained in aggression chains. Such signals are then accessible to individuals capable of transitive inference.

We also evaluated evidence for the use of depth-based measures of rank, where individuals are evaluated based not only on the aggression they receive, but on the characteristics of their aggressors. In particular, weighted rank difference, *W*(*n*), allows us to investigate whether social information was encoded within smaller subsets of the total aggression network in chains of aggression (Fig 4). We refer to this rank-inference method as the “motif pathway”.

In the null (dashed line in Fig 4), we find that chain length encodes little or no information about relative rank. Individuals tend to be lower-ranked than their aggressors, but seeing an individual at the end of a long chain provides little or no additional information about its rank. By contrast, the observed data (solid line in Fig 4) encoded significant information in aggression chains. In both Groups One and Two, local motifs contained a substantial amount of global information.

An observation of an individual at the end of a longer chain (length ≥ 3) provided more information about relative rank than an observation of an individual at the end of a short chain (length 1 or 2). This encoding potentially allowed individuals to distinguish between individuals nearby (Δ ∼ 5) and distant (Δ > 10) in rank—a discrimination impossible in the nulls.

This is our second main result: network motifs, in addition to breadth-based magnitude measures, can provide signals of relative rank.

**Fig 5:**
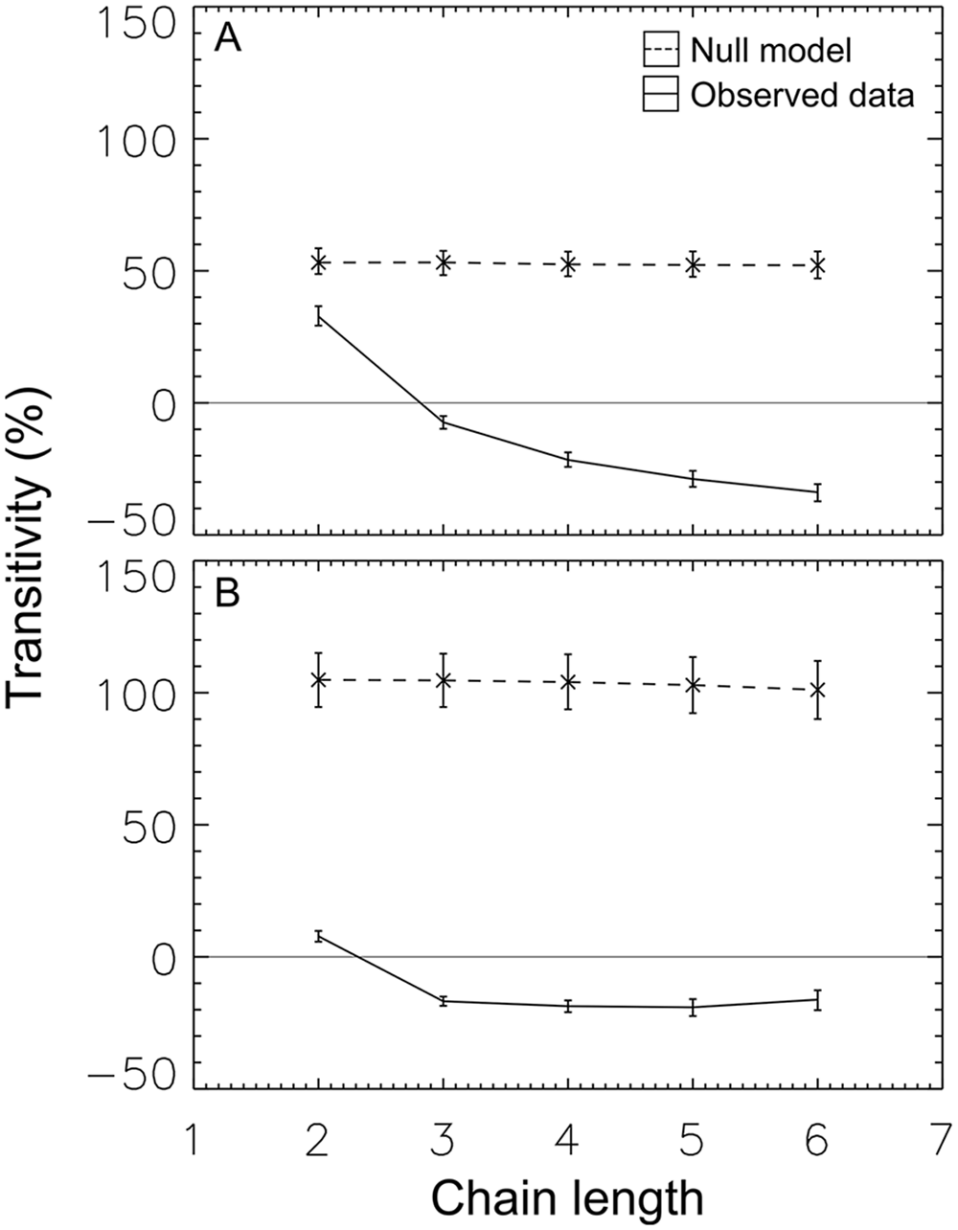
Aggression chains predict behavioral preferences. In both Group One (a) and Group Two (b), aggression against those at the end of a chain was reduced compared to null. For aggression chains three steps or longer, we find preference inversion: negative transitivity, meaning that (on average) *i* directed less-than-average aggression against *l* if *l* was found at the end of a chain *i* → *j* → *k* → *l*.

Fractional transitivity, *T*(*n*), allows us to investigate whether these aggression chains are predictive of actual behavior. Our analysis found a strong difference between the null model and the observed data (Fig 5). In the null (dashed line), chains predict increased aggression: if *i* aggresses against *j*, and *j* against *k*, this leads *i*, on average, to direct increased aggression against *k*. This is independent of chain length—both long and short chains predict similar levels of increased aggression.

In contrast, behavior in the observed data (Fig 5, solid line) showed the opposite effect. While short chains predicted a (small amount) of increased aggression (*T*(2) greater than zero), long chains were associated with reduced aggression (*T*(*n*) less than zero for *n* greater than two). This remarkable inversion is what is expected when individuals use the information content of motifs to predict relative rank, and then both (1) preferentially avoid conflict with much lower-ranked individuals and (2) focus aggression on rank neighbors.

This is our third main result: network motifs predict behavior.

### Quarter-by-quarter dynamics of *W*(*n*) and *T*(*n*)

Having demonstrated the existence and predictive power of signals, we can investigate the time-frame over which the signals themselves emerge by looking at *W*(*n*) quarter-by-quarter. In Group One we can already find evidence for the existence of these signals, over and above null expectation, at the one-step and two-step level, in the first quarter. The signals were present, but *R*(Δ) results suggest that they are not yet used by participants. By contrast, the signal is almost entirely absent in the first quarter for Group Two. After the first week (*i.e.*, for the later quarters) the signal became significantly stronger, covered a wider range of ranks, and we saw signals at the three-step, and often at the four-step, level in both groups (S7 Fig and S8 Fig).

We can also examine *T*(*n*) quarter-by-quarter to look for the dynamical emergence of behavioral preference. The first quarter showed some evidence for the use of motif information to structure behavior, with shifts in preferences of on average 32 percentage points relative to the null in both groups. Effects became larger at later times, with behavioral preferences shifting by on average 82 percentage points relative to the null in the final three quarters. Preference inversion appears only in these later quarters. The emergence of this behavioral pattern parallels the emergence, over time, of the large scale order seen in Fig 2, and of cognitively-accessible signals seen in Fig 4 (S9 Fig and S10 Fig).

## Discussion

Our results show how group social structure forms, how cognitively-accessible signals allow for inference of rank, and how these signals predict core features of actual behavior in captive parakeet groups. Our study provides insight into two longstanding questions about dominance in complex groups: how does dominance emerge, and how do individuals infer and act upon it?

We found that (1) a transition towards more structured aggression occurred rapidly, about a week after initial group formation, (2) behavioral motifs and chains of aggression provided information about relative rank, and (3) symbolic distance along aggression chains was predictive of aggression preferences. We found that use of the magnitude pathway, WSC, could only explain some of this structure, but that individuals could supplement this with depth-based information in the form of aggression chains.

The patterns seen in Fig 3 are thus driven, on the one hand, by information about rank (in both the magnitude pathway, and the motif pathway seen in Fig 4), and, on the other hand, by decisions to aggress that we track in Fig 5. We have found that both the motif signal, and the extent to which it predicts aggression patterns, increase over time, paralleling the emergence of the large-scale order seen in Fig 3. Importantly, we found that dominance hierarchy structure emerges despite the absence of visual or spatial cues to rank. Furthermore, the speed with which this structured behavior emerges means that “conventional” visual cues—physical markings that serve as badges of status not logically related to fighting ability [34]—would not have had time to develop.

Our results thus imply the existence of a feedback between behavior and knowledge. Aggression at the individual level leads to large-scale knowledge about dominance rank at the group level. Individuals are able to gain knowledge about these ranks, and to use this information to adjust their behavior accordingly.

Over time, this leads to the emergence of global social signals, including the dominance ranks that have been a central part of study in animal behavior. This feedback loop is dependent on individuals possessing two critical cognitive skills: social memory and social inference. Individuals must be able to identify individuals and remember the outcomes of past events, and then integrate these memories to structure subsequent behavior. This feedback loop may play a key role in dominance hierarchy development in larger, more complex social groups.

We focused on two distinct knowledge pathways: (1) the magnitude pathway, which requires observers to track incoming aggression to each individual, regardless of source identity and (2) the motif pathway, which requires partial knowledge of aggression network structure.

Under the magnitude pathway, the number of aggressors and the total amount of aggression targeted at each individual is tracked to determine rank. As more interactions occur, more information exists upon which rank can be inferred. Although the magnitude pathway is more likely to be cognitively accessible to individuals [75], decisions made on the basis of this pathway alone are unable to explain all of the behavior we see. Depth-based measures provide additional discriminatory power, and are predictive of actual aggression including the rank-focused aggression seen in Fig 3.

Once these signals are in place, effects are strong. Aggression levels differed by a factor of two or more from the null model (Fig 3), signals allowed individuals to discriminate across nearly the full range of relative ranks (Fig 4), and these signals predict shifts in behavioral preferences of 50 percentage points or more (Fig 5).

Significant features of the social signal are contained in the two- and three-step aggression chains. That observed shifts in behavior (quantified by *T*(*n*)) can be due to direct reliance on the signal (detected by *W*(*n*)) is supported by experimental studies. These find that larger symbolic distances are not only salient, but actually more easily perceived than neighboring pairs [83, 84]. Meanwhile, our detection of rank focused aggression is consistent with results that find nearby ranks perceptually salient, including the finding that rank reversals occurring at small relative rank differences are perceived as more stressful [45].

Spatial assortment may be a signal of rank in some systems [39]. However, we found no evidence that parakeets use either spatial assortment or physical size to structure their aggression. Instead, our work suggests that the formation of a hierarchy relies on cognitive inference over complex relationships. Our work has found that aggression motifs contain a substantial amount of information that could be used infer relative rank.

Despite being more cognitively demanding, the motif pathway explains structuring of aggression over and above that from the magnitude pathway. Use of this signal requires that individuals reason inductively about relationships over and above observed pairwise comparisons, an ability known as transitive inference. While transitive inference (*i* beats *j*, *j* beats *k*, therefore *i* beats *k*) is more sophisticated than recall of pairwise comparisons (*i* beats *j*), experimental work has established that a wide range of social species have the ability to infer or learn indirectly [85]. Evidence for transitive inference has been documented in both non-social [83, 86, 87] and social contexts [88–90]. Meanwhile, observational studies find evidence for the use of higher-order (*i.e.*, beyond pairwise) strategies where actions of one individual against another are influenced by third parties, both in individual and group-level decision-making and perception [48, 49, 91–93]. Reliance on transitive motifs provides a natural source of such rules: a decision to aggress against a target can be influenced by the aggression that others display against the target.

Previous work has shown how relatively simple rules can be used to govern dyadic or triadic interactions at a local scale. Here, we have considered how hierarchies form on larger scales in more complex social groups. As group size increases, the number of relationships that must be tracked to determine group-level rankings increases dramatically. Our results here help explain how this complexity can be managed by use of cognitively-accessible pathways, and how the use of such pathways may feed back to how individuals choose to aggress.

The question of cognitive complexity is crucial. A human observer equipped with sophisticated computational tools can determine rank order given knowledge of all pairwise aggressive events. However, it is highly unlikely that the individuals have the cognitive ability needed to use these methods, and they must rely in part on simpler, “ecological” [94–96] methods such as the magnitude and motif pathways described above.

The ability to construct a model of the dominance hierarchy, based on a combination of direct experience and indirect observation of others, could be particularly adaptive in species where dispersal results in the regular integration of new members into existing groups and in groups with high fission-fusion dynamics. Reliance on, and consequent amplification of, cognitively-accessible signals means that individuals would not have to directly interact with all possible combinations of individuals in the group.

Indirect inference of dominance rank can allow individuals to predict the behavior of others while conserving energy and reducing the possibility of injury [97]. It can also facilitate integration of immigrants into existing groups, and allow more rapid formation of dominance hierarchies. This may be particularly adaptive in species where dispersal results in the regular integration of new members into existing groups, and in groups with high fission-fusion dynamics.

### Conclusions

How individuals come to know their social worlds, and how that knowledge feeds back to influence social properties, is a crucial part of group dynamics. This paper has tracked the emergence of strategically directed aggression, the signals that could enable it, and how these signals predict decisions to aggress.

Previous work on transitive inference by non-human animals has often focused on experimental manipulation of trained subjects [83, 85]. These experiments are generally removed from social situations. They can provide critical evidence that individuals have the necessary cognitive skills, but usually cannot show a direct link between those abilities and an understanding of the social landscape of an actual group and its effect in real-world situations. Our work provides new evidence for the importance of transitive inference in the real-world problem of directing aggression.

Our findings on the information contained in chains of aggression and the strategic use of this information allow us to construct a mechanistic account of both what signals of rank in observed behavior might be available to individuals (the knowledge pathway) and how these signals influence decision-making (the behavior pathway). It allows us to explain the dynamical transition as the onset of a complex interaction between knowledge of rank and consequent behavior. In the closing of this knowledge-behavior feedback loop are the seeds of complex society.

## Supporting Information

S1 Table (PDF)

S1 Text (PDF)

S2 Text (PDF)

S3 Text (PDF)

S4 Text (PDF)

S1 Fig (PDF)

S2 Fig (PDF)

S3 Fig (PDF)

S4 Fig (PDF)

S5 Fig (PDF)

S6 Fig (PDF)

S7 Fig (PDF)

S8 Fig (PDF)

S9 Fig (PDF)

S10 Fig (PDF)
