# Supplementary material for: Social Feedback and the Emergence of Rank in Animal Society
Source: PLoS Comput Biol. 2015 Sep 10;11(9):e1004411. doi: 10.1371/journal.pcbi.1004411 (PMC4565698; doi:10.1371/journal.pcbi.1004411)
Supplement: S1 Table — (PDF) [file pcbi.1004411.s001.pdf]

Supporting Information:
Social Feedback and the Emergence of Rank in Animal Society

Elizabeth A. Hobson & Simon DeDeo

S1 Table. Correlations between Eigenvector Centrality and other measures. Correlations of power scores (in the case of David's Score and WSC) and overall rank (in the case of I&SI, which provides only an ordering, not a score). In all cases, correlations are significant at $p < 0.001$. Analysis is for the final three quarters of the data. Eigenvector Centrality (EC) is our main way to characterize the dominance hierarchies of both our systems.

	Group One	Group Two	Type
EC to David's Score	0.96	0.95	Pearson r^2
EC to WSC	0.73	0.73	Pearson r^2
EC to I&SI	0.93	0.76	Spearman
