# Supplementary material for: Social Feedback and the Emergence of Rank in Animal Society
Source: PLoS Comput Biol. 2015 Sep 10;11(9):e1004411. doi: 10.1371/journal.pcbi.1004411 (PMC4565698; doi:10.1371/journal.pcbi.1004411)
Supplement: S1 Text — (PDF) [file pcbi.1004411.s002.pdf]

# Supporting Information:
# Social Feedback and the Emergence of Rank in Animal Society

Elizabeth A. Hobson & Simon DeDeo

### S1 Text. Average Preferential Rank Aggression

Rather than quantify the total aggression in the system (Average Rank Aggression; see Eq. 3, main text), we can also measure individual-level preferences. We can quantify the average fraction of an individual's aggression that is directed $\Delta$ steps away in the hierarchy by measuring $R_{\mathrm{pref}}(\Delta)$, defined as

$$R_{\mathrm{pref}}(\Delta) = \frac{1}{N_\Delta} \sum_{i=1}^{N} \frac{d_{i\Delta}}{d_i}, \tag{1}$$

where $d_i$ is the total aggression seen for individual $i$. If $R(2)$ is 0.1, for example, it means that, on average, an individual directs 10% of their aggression towards an individual two steps down in the hierarchy.
