# Supplementary material for: Social Feedback and the Emergence of Rank in Animal Society
Source: PLoS Comput Biol. 2015 Sep 10;11(9):e1004411. doi: 10.1371/journal.pcbi.1004411 (PMC4565698; doi:10.1371/journal.pcbi.1004411)
Supplement: S2 Text — (PDF) [file pcbi.1004411.s003.pdf]

Supporting Information: Social Feedback and the Emergence of Rank in Animal Society

Elizabeth A. Hobson & Simon DeDeo

S2 Text. Null models for aggression. To compute power, EC maps the pattern of directed aggression to a Markov process. We define the matrix $\mathbf{d}$, where an element d_{ij} is the number of times individual i aggressed against j. From $\mathbf{d}$, we can calculate the transition matrix $\mathbf{t}$

$$t_{ij} = \frac{d_{ij} + \epsilon}{\sum_{k=1}^{n} (d_{ik} + \epsilon)}, \tag{1}$$

where t_{ij} is the probability that if i is observed aggressing, it is aggressing against j, and ϵ is a small regularizing term. The power distribution, v, is the stationary distribution of this transition matrix; equivalently, the dominant left-eigenvector of the matrix $\mathbf{t}$.

There are $n(n-1)$ free parameters in an aggression network, but only $n-1$ integers are required to define a linear ranking system. Thus, any ranking system amounts to a lossy compression of the original data, summarizing the behavioral patterns relevant to the establishment of a dominance hierarchy. Conversely, for any given dominance hierarchy there are many possible behavioral patterns. Our null model for aggression is defined as sampling randomly from this set.

In particular, because there are many possible $\mathbf{d}$ and $\mathbf{t}$ matrices compatible with a particular power distribution v, we can define a null model as random draws from the set of matrices that have, on average, the same v. Now we constrain not only the linear ranking, but also a power-score.

We construct a measure over this set implicitly, sampling from by means of the following algorithm.

1. Take k is equal to $\sum_{ij} d_{ij}$, *i.e.*, the total number of observed aggressions in the data.

2. Construct a k-element time-series, T, of individuals, $\sigma_1\sigma_2\ldots\sigma_k$, where the σ_i are drawn by means of iid samples from $E_1(\mathbf{d})$.

3. Re-order T so that it contains no consecutive pairs, *i.e.*, so that $\sigma_i \neq \sigma_{i+1}$.

4. Use the Expectation-Maximization (EM) algorithm [1] to find the transition matrix $\mathbf{t}'$ for a Markov Model consistent with this time-series. Since t_{ii} is equal to zero, our reordering will fix t_{ii} equal to zero and the final solution $\mathbf{t}'$ found by the EM algorithm respects this constraint.

5. Convert $\mathbf{t}'$ to an aggression series $\mathbf{d}'$ by fixing $d_{ij} = t_{ij} \sum_j d'_{ij}$.

The time-series $\{\sigma_i\}$ itself can be interpreted as a linked chain of observed aggression pairs, $\sigma_i \to \sigma_{i+1}$, $\sigma_{i+1} \to \sigma_{i+2}$, and so forth, but this time series should not be interpreted as a potential observation; correlations *between* aggressive acts in the series are not constrained to those observed in the real world.

A particular null transition matrix $\mathbf{t}'$ represents aggression preferences, which we then map to a set of expected behaviors, $\mathbf{d}'$, or a set of counts that could have been observed in the captive groups. We assume the same overall aggression levels for each individual seen in the original data *i.e.*, d'_{ij} is equal to $t'_{ij} \sum_j d_{ij}$. We generated 10,000 null replicates.

In our figures reporting $R(\Delta)$ we bin neighboring ranks (*i.e.*, we estimate $R(\Delta)$ by averaging the raw measurements for $\Delta - 1$, Δ and $\Delta + 1$, reflects RMS uncertainties in the ranks themselves; when Δ is equal to one, we average 1 and 2, and similarly for Δ equal to negative one. This improves signal to noise in each bin at the cost of correlating bins. To determine errors in the observed data (red bands in our Figures 2 and 3 in the main paper), we use the statistical bootstrap and resample with replacement from the original observed data, reporting median and 1σ bands. As in the case of the null model, we bin neighboring ranks.

References

1. Press WH. Numerical Recipes: The Art of Scientific Computing. Cambridge, UK: Cambridge University Press; 2007. 3rd Edition.
