# Supplementary material for: Social Feedback and the Emergence of Rank in Animal Society
Source: PLoS Comput Biol. 2015 Sep 10;11(9):e1004411. doi: 10.1371/journal.pcbi.1004411 (PMC4565698; doi:10.1371/journal.pcbi.1004411)
Supplement: S3 Text — (PDF) [file pcbi.1004411.s004.pdf]

Supporting Information:
Social Feedback and the Emergence of Rank in Animal Society

Elizabeth A. Hobson & Simon DeDeo

S3 Text. Definitions of $W(n)$ and $T(n)$.

As in the main text, we defined the weighted rank aggression as

$$W(2) = \frac{\sum_{i,j,k;\emptyset} d_{ij} d_{jk} \Delta(i,k)}{\sum_{i,j,k;\emptyset} d_{ij} d_{jk}} \tag{1}$$

where we write $\emptyset$ to indicate the restriction to distinct values of i, j, and k. Higher orders can be defined in a similar fashion, so that

$$W(3) = \frac{\sum_{i,j,k,l;\emptyset} d_{ij} d_{jk} d_{kl} \Delta(i,l)}{\sum_{i,j,k,l,m;\emptyset} d_{ij} d_{jk} d_{kl}}, \tag{2}$$

and

$$W(4) = \frac{\sum_{i,j,k,l,m;\emptyset} d_{ij} d_{jk} d_{kl} d_{lm} \Delta(i,m)}{\sum_{i,j,k,l,m;\emptyset} d_{ij} d_{jk} d_{kl} d_{lm}}, \tag{3}$$

and so forth, always restricting to non overlapping choices for the indices.

Similarly, the transitivity is defined as

$$T(2) = \frac{\sum_{i,j,k;\emptyset} d_{ij} d_{jk} (d_{ik} - \bar{d}_{i;j})/\bar{d}_{i;j}}{\sum_{i,j,k;\emptyset} d_{ij} d_{jk}}, \tag{4}$$

where $\bar{d}_{i;j}$ is the average aggression directed by i against individuals other than j. We exclude j from this average so as not to induce a spurious correlation. and higher orders can be defined as

$$T(3) = \frac{\sum_{i,j,k,l;\emptyset} d_{ij} d_{jk} d_{kl} (d_{il} - \bar{d}_{i;j})/\bar{d}_{i;j}}{\sum_{i,j,k,l;\emptyset} d_{ij} d_{jk} d_{kl}}, \tag{5}$$

and

$$T(4) = \frac{\sum_{i,j,k,l,m;\emptyset} d_{ij} d_{jk} d_{kl} d_{lm} (d_{im} - \bar{d}_{i;j})/\bar{d}_{i;j}}{\sum_{i,j,k,l,m;\emptyset} d_{ij} d_{jk} d_{kl} d_{lm}}, \tag{6}$$

and so forth, always restricting to non overlapping choices for the indices.
