# Supplementary material for: Social Feedback and the Emergence of Rank in Animal Society
Source: PLoS Comput Biol. 2015 Sep 10;11(9):e1004411. doi: 10.1371/journal.pcbi.1004411 (PMC4565698; doi:10.1371/journal.pcbi.1004411)
Supplement: S4 Text — (PDF) [file pcbi.1004411.s005.pdf]

Supporting Information:
Social Feedback and the Emergence of Rank in Animal Society

Elizabeth A. Hobson & Simon DeDeo

S4 Text. Weighted Simple Consensus

Ref. [1] described a simple, cognitively accessible measure of network consensus called Weighted Simple Consensus. Under WSC, an individual's score is the product of the total amount of aggression (number of displacements) received, multiplied by the number of distinct individuals who directed that aggression. WSC power correlates strongly with EC power in both groups (r^2=0.73 for the final three quarters, in both groups; r^2=0.70 quarter-by-quarter average), and because of its relative simplicity may thus play the role of a signal of relative rank—in particular, individuals may use WSC to estimate relative rank, and use those estimates to guide their decision-making.

If WSC does indeed play the role of a rank indicator that individuals use to direct aggression, we should see an imprint of this decision-making in the data itself; in particular, we should see that average aggression is focused, over and above the null, as a function of WSC rank. Indeed, this is what is found in both groups, as shown in S6 Fig.: we find strong, above null signals that relative rank, when estimated by WSC, predicts focused aggression, most obviously in the suppression of long-range rank aggression.

Comparing S6 Fig. and Fig. 3 of the main paper, we see that, while (1) WSC is a predictor of EC rank, and (2) WSC is correlated with rank-focused behavior, it is also the case that rank focusing is much stronger under the EC measure than under WSC. In group one, for example, rank aggression directed at rank separations less than five is a factor of 1.95 above null when using EC ranks, but only 1.34 above null when using WSC ranks; meanwhile, in both group one and group two, there is greater suppression of long-range rank aggression when using EC ranks compared to WSC ranks.

Given this greater focusing, if individuals were using WSC directly, it would require unusual fine-tuning for them to pattern their usage to achieve a tighter focus in EC than they do in WSC itself. This leads us to consider the possibility, in the main paper, that individuals may also rely on depth-based measures of relative rank.

References

1. Brush ER, Krakauer DC, Flack JC. A Family of Algorithms for Computing Consensus about Node State from Network Data. PLoS Computational Biology. 2013;9(7):e1003109.
