# Supplementary material for: Social Feedback and the Emergence of Rank in Animal Society
Source: PLoS Comput Biol. 2015 Sep 10;11(9):e1004411. doi: 10.1371/journal.pcbi.1004411 (PMC4565698; doi:10.1371/journal.pcbi.1004411)
Supplement: S1 Fig — (PDF) [file pcbi.1004411.s006.pdf]

Supporting Information:
Social Feedback and the Emergence of Rank in Animal Society

Elizabeth A. Hobson & Simon DeDeo

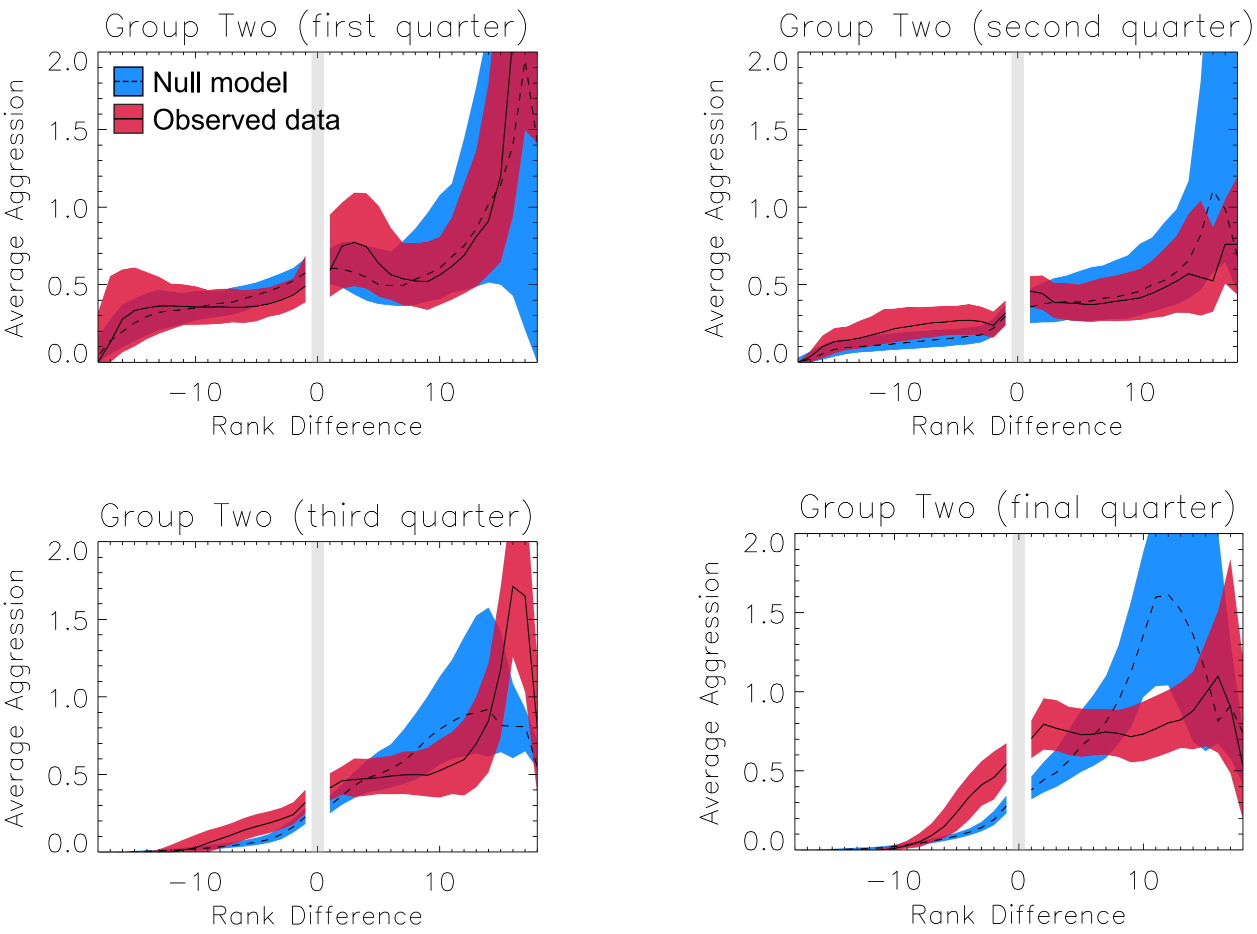

S1 Fig. Onset of structured aggression with NBB included. As described in the main text, we removed one anomalous individual (NBB) from our analyses of Group Two; here, for completeness, we show the late-time stationary properties of Group Two including NBB. Immediately apparent is a peak in the average aggression directed towards much lower ranks. Including NBB in the analysis of Group Two leads to an apparent delay in the onset of structured aggression as aggression against NBB drives it down the hierarchy.
