# Supplementary material for: Social Feedback and the Emergence of Rank in Animal Society
Source: PLoS Comput Biol. 2015 Sep 10;11(9):e1004411. doi: 10.1371/journal.pcbi.1004411 (PMC4565698; doi:10.1371/journal.pcbi.1004411)
Supplement: S2 Fig — (PDF) [file pcbi.1004411.s007.pdf]

Supporting Information:
Social Feedback and the Emergence of Rank in Animal Society

Elizabeth A. Hobson & Simon DeDeo

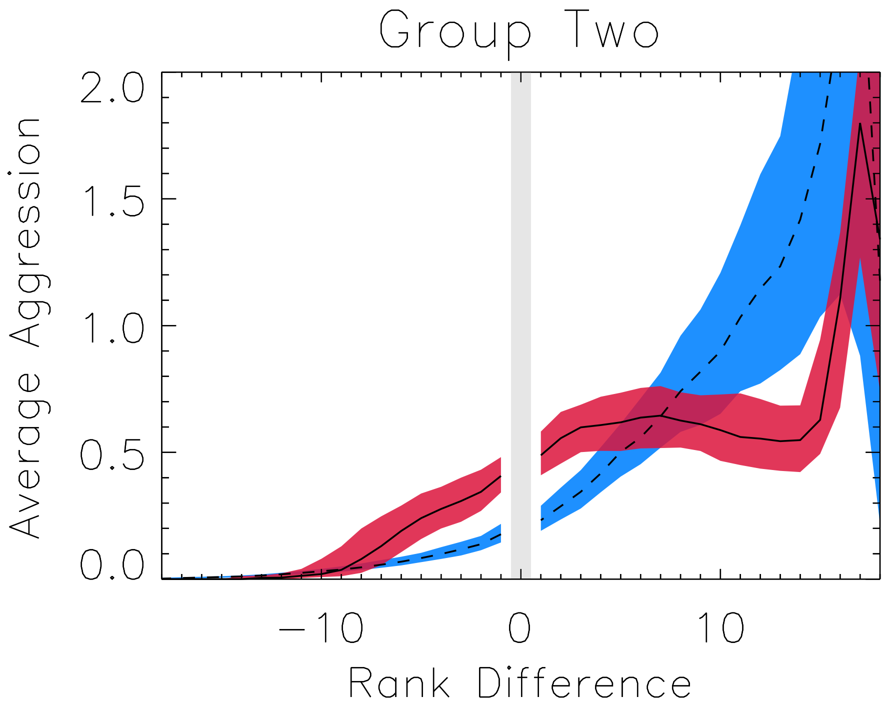

S2 Fig. Stationary features of Group Two with NBB included. Stationary features of Group Two are largely unchanged by the inclusion of NBB, except for the peak at the largest rank differences corresponding to aggression directed against the lowest ranking individual, NBB, by high-ranking individuals who repel NBB's unwanted attempts at affiliation. Multi-modal observations allow us to attribute these effects entirely to NBB's anomalous attempts to affiliate with high-ranking individuals who continually repelled these unwanted attempts. Exclusion of this single individual completely eliminates this peak, as can be seen in Fig. **??** in the Main Text. Inclusion of NBB also leads to an apparent delay in the onset of structured aggression in Group Two.
