# Supplementary material for: Social Feedback and the Emergence of Rank in Animal Society
Source: PLoS Comput Biol. 2015 Sep 10;11(9):e1004411. doi: 10.1371/journal.pcbi.1004411 (PMC4565698; doi:10.1371/journal.pcbi.1004411)
Supplement: S3 Fig — (PDF) [file pcbi.1004411.s008.pdf]

Supporting Information:
Social Feedback and the Emergence of Rank in Animal Society

Elizabeth A. Hobson & Simon DeDeo

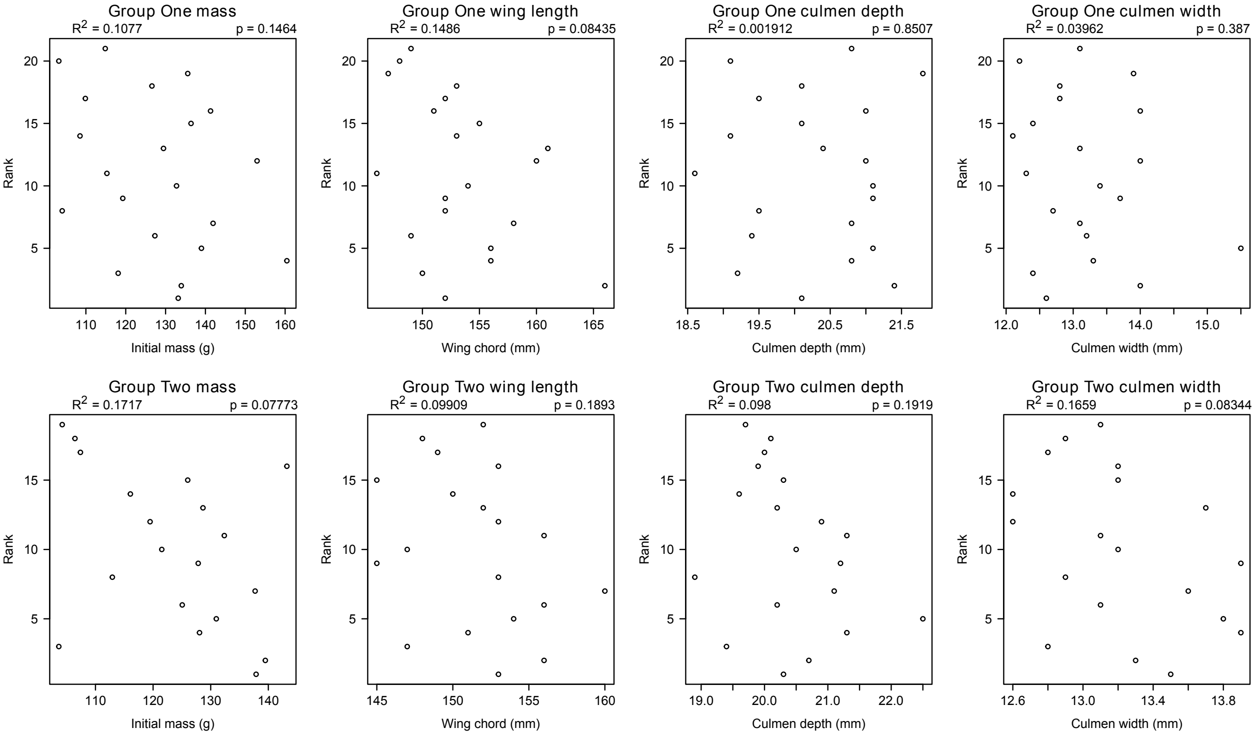

S3 Fig. Physical characteristics do not signal rank. We looked for correlations between four main physical cues—mass, wing length, culmen depth and culmen width—and rank. In all four cases, and in both groups, we found no evidence of any rank signal. Correlations were close to zero, and consistent with the null, each case. (Culmen measurements refer to the size of the beak; depth and width are both measured at the nares, which is roughly the base of the beak.) We find no evidence that rank could be reliably determined based on simple underlying cues such as size or spatial proximity. None of the morphometric body size measures provided any rank signal in either of the two study groups. Rank was not significantly associated with the physical size of individuals, including weight, wing length, and beak properties ($|r^2| < 0.18$, $p > 0.05$).
