# Supplementary material for: Social Feedback and the Emergence of Rank in Animal Society
Source: PLoS Comput Biol. 2015 Sep 10;11(9):e1004411. doi: 10.1371/journal.pcbi.1004411 (PMC4565698; doi:10.1371/journal.pcbi.1004411)
Supplement: S4 Fig — (PDF) [file pcbi.1004411.s009.pdf]

Supporting Information:
Social Feedback and the Emergence of Rank in Animal Society

Elizabeth A. Hobson & Simon DeDeo

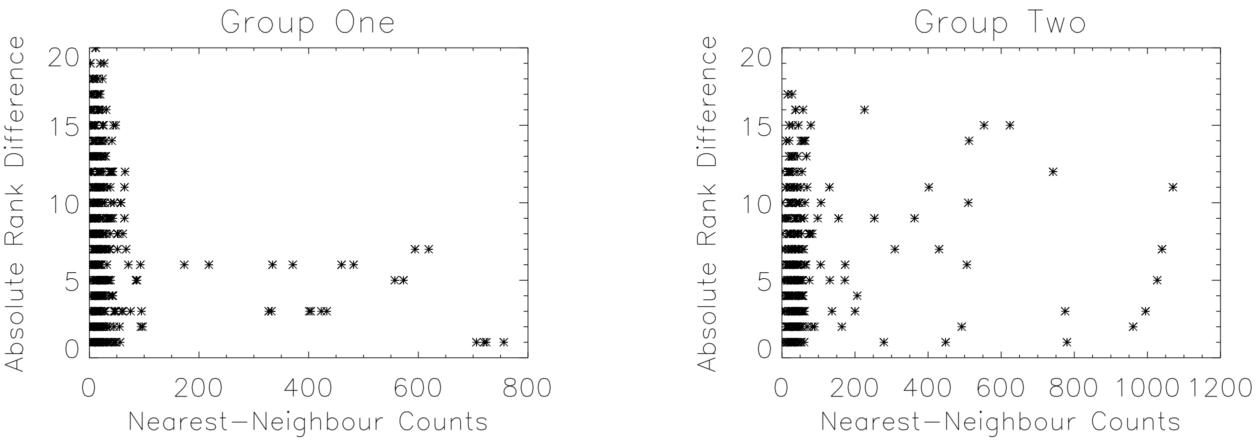

S4 Fig. Neighbor identity is not a strong signal of rank. Spatial proximity (via neighbor identity) is not a strong signal of rank in either Group One ($r^2 = -0.12$) or Group Two ($r^2 = -0.02$). Even in the final three-quarters, where behavior is most regular, in Group One, proximity provides only a weakly anticorrelated signal ($r^2 = -0.14$); in Group Two, it provides no signal at all ($r^2 = 0.04$; consistent with null). Note that each point here represents a pair; there are thus 210 points in Group One and 171 points in Group Two on these two plots, with the vast majority of points overlapping near zero on the x axis.
