# Supplementary material for: Social Feedback and the Emergence of Rank in Animal Society
Source: PLoS Comput Biol. 2015 Sep 10;11(9):e1004411. doi: 10.1371/journal.pcbi.1004411 (PMC4565698; doi:10.1371/journal.pcbi.1004411)
Supplement: S5 Fig — (PDF) [file pcbi.1004411.s010.pdf]

Supporting Information:
Social Feedback and the Emergence of Rank in Animal Society

Elizabeth A. Hobson & Simon DeDeo

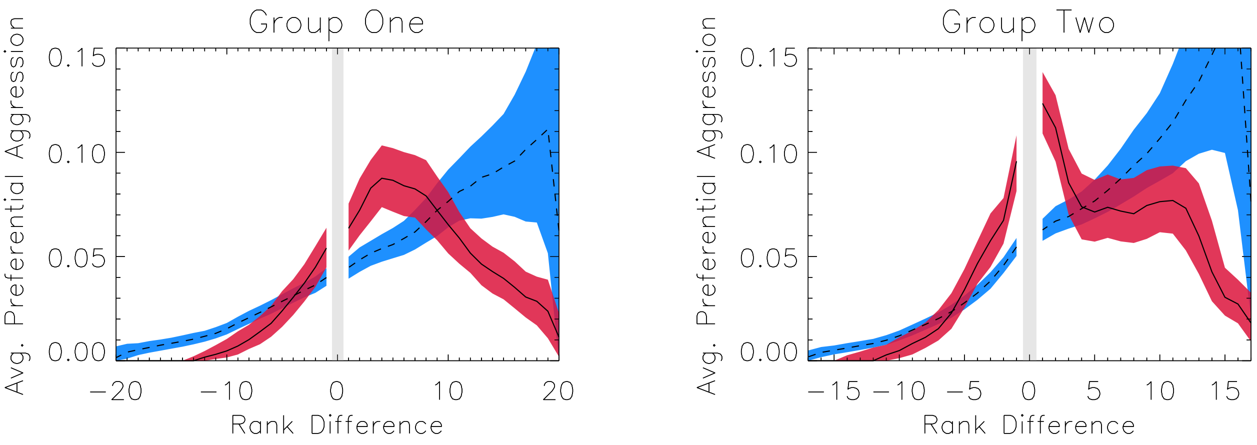

S5 Fig. Average Preferential Rank Aggression. $R_{\text{pref}}(\Delta)$ for Groups One and Two (these plots can be directly compared to Fig. 3 of the main text). We see the same phenomenon of both rank focusing for nearby ranks and reduced aggression towards those further down in the hierarchy. In Group One, aggression preferences towards individuals one to five ranks downwards in the hierarchy is 59% higher than null ($p < 0.001$); in Group Two, preferences are 31% higher than null ($p < 0.001$). We also see the same evidence for the *emergence* of this rank-focusing pattern. In the first quarter, we find no evidence of elevated rank-focused aggression in either Group One or Group Two ($p > 0.1$). In quarters two, three, and four, by contrast, we see strong evidence for rank focusing both groups (48%, 79% and 98% increases, quarter-by-quarter in Group One, $p < 0.001$; 45% ($p < 0.001$), 59% ($p < 0.001$), and 23% ($p < 0.05$) in Group Two).
