# Supplementary material for: Social Feedback and the Emergence of Rank in Animal Society
Source: PLoS Comput Biol. 2015 Sep 10;11(9):e1004411. doi: 10.1371/journal.pcbi.1004411 (PMC4565698; doi:10.1371/journal.pcbi.1004411)
Supplement: S6 Fig — (PDF) [file pcbi.1004411.s011.pdf]

Supporting Information:
Social Feedback and the Emergence of Rank in Animal Society

Elizabeth A. Hobson & Simon DeDeo

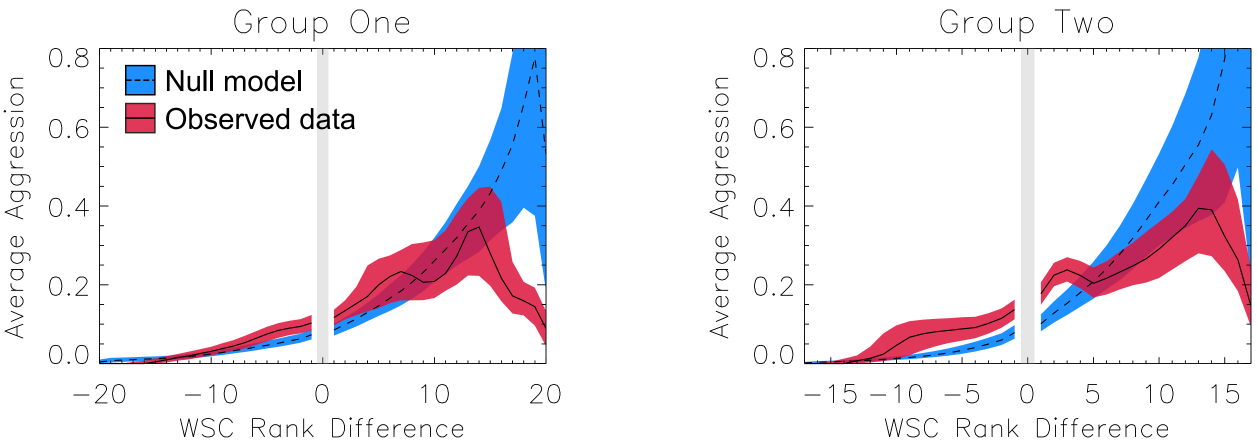

S6 Fig. Weighted Simple Consensus as a guide to focused aggression. Aggression patterns plotted for the final three study quarters. Red band is average aggression as a function of rank difference (and 1σ errors); blue is the EC null as before.
