# Supplementary material for: Social Feedback and the Emergence of Rank in Animal Society
Source: PLoS Comput Biol. 2015 Sep 10;11(9):e1004411. doi: 10.1371/journal.pcbi.1004411 (PMC4565698; doi:10.1371/journal.pcbi.1004411)
Supplement: S7 Fig — (PDF) [file pcbi.1004411.s012.pdf]

Supporting Information: Social Feedback and the Emergence of Rank in Animal Society

Elizabeth A. Hobson & Simon DeDeo

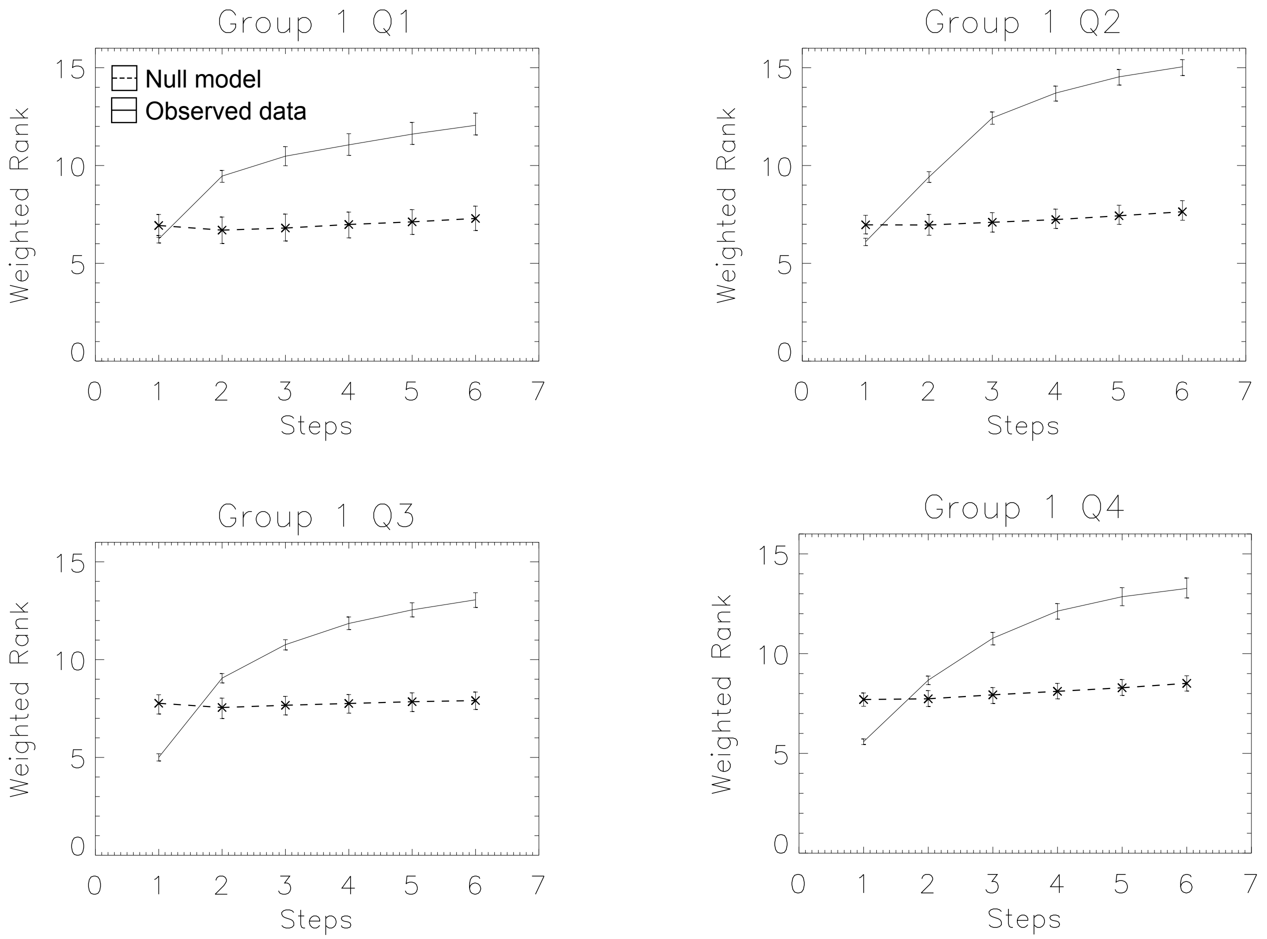

S7 Fig. $W(n)$ **broken out quarter-by-quarter for Group One.** Transitive signals of rank already appear in Quarter One, where the one- and two-step aggression chains can clearly distinguish relative ranks. In Quarters Two, Three and Four, these signals strengthen, so that three- and even four-step aggression chains now contain additional information.
