# Supplementary material for: Social Feedback and the Emergence of Rank in Animal Society
Source: PLoS Comput Biol. 2015 Sep 10;11(9):e1004411. doi: 10.1371/journal.pcbi.1004411 (PMC4565698; doi:10.1371/journal.pcbi.1004411)
Supplement: S8 Fig — (PDF) [file pcbi.1004411.s013.pdf]

Supporting Information:
Social Feedback and the Emergence of Rank in Animal Society

Elizabeth A. Hobson & Simon DeDeo

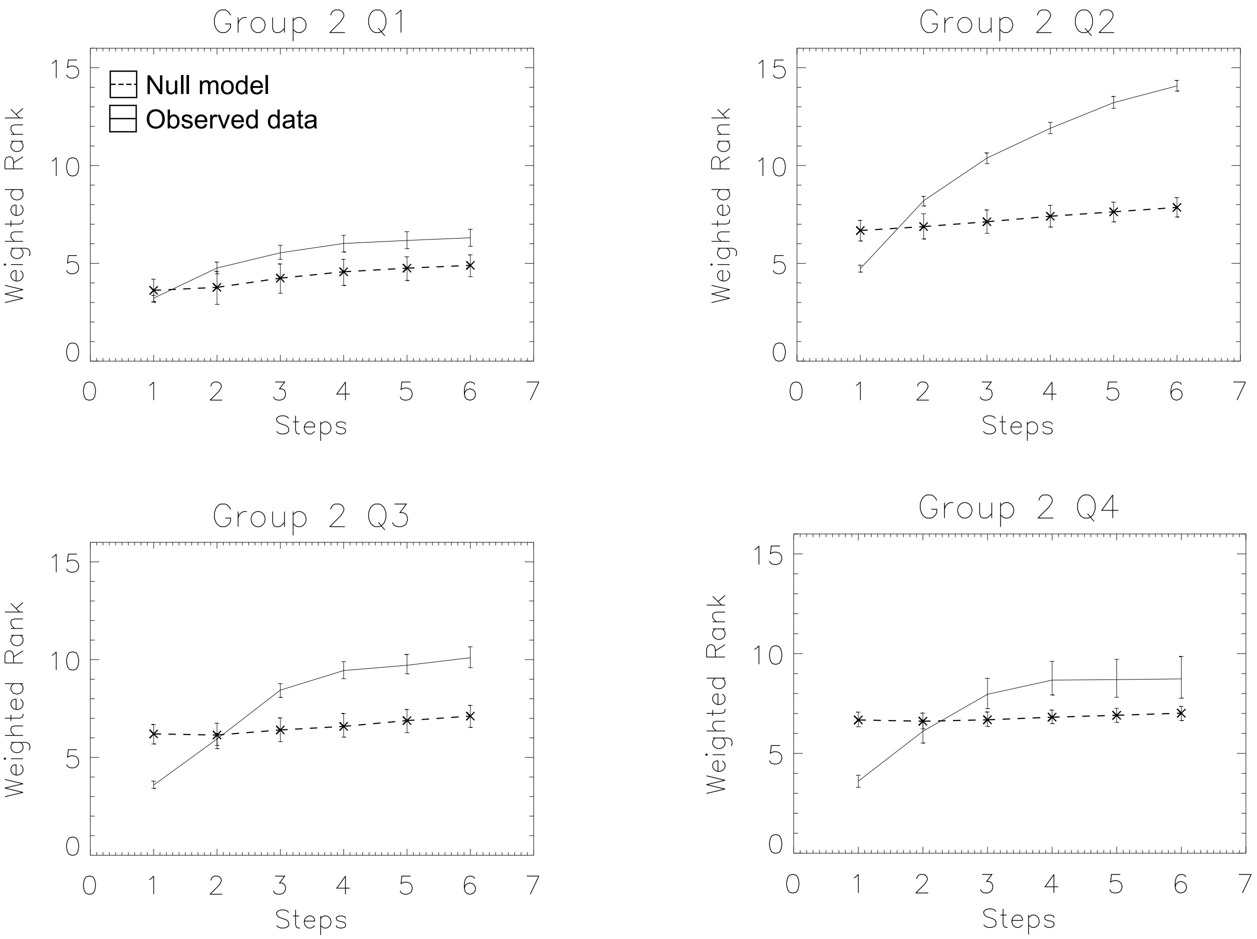

S8 Fig. $W(n)$ **broken out quarter-by-quarter for Group Two.** Very little information exists in Quarter One; In Quarters Two, Three and Four, chains up to length three and even four (in Quarter Two) contain distinct information. In both Groups One and Two, signals are much stronger, and remain much stronger, after the first week, with Quarters Two, Three, and Four in both groups having significant information of relative rank in the one-, two- and three-step chains, and (to a more limited extent) in the four-step chains.
