# Supplementary material for: Social Feedback and the Emergence of Rank in Animal Society
Source: PLoS Comput Biol. 2015 Sep 10;11(9):e1004411. doi: 10.1371/journal.pcbi.1004411 (PMC4565698; doi:10.1371/journal.pcbi.1004411)
Supplement: S10 Fig — (PDF) [file pcbi.1004411.s015.pdf]

Supporting Information:
Social Feedback and the Emergence of Rank in Animal Society

Elizabeth A. Hobson & Simon DeDeo

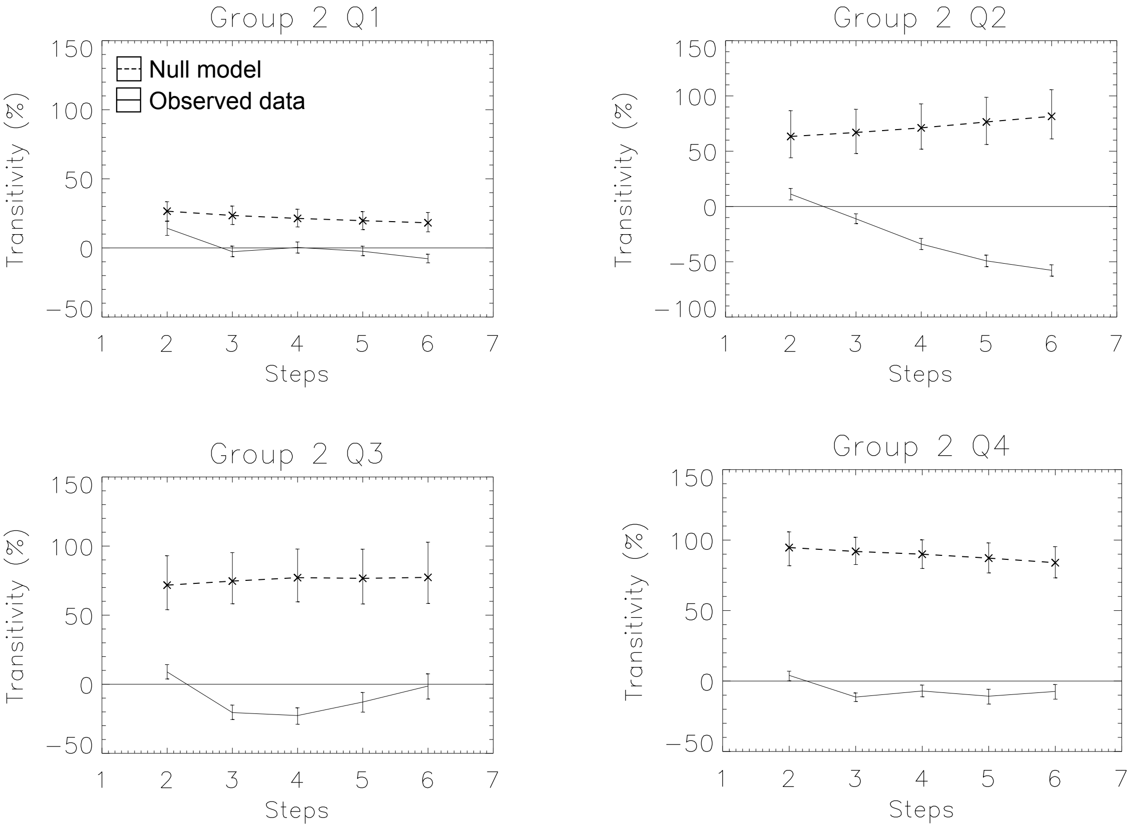

S10 Fig. $T(n)$ **broken out quarter-by-quarter for Group Two.**
